# Editorial: Subtypes of typical migraine with aura: exploring markers for subtype classification and treatment response

**DOI:** 10.3389/fnhum.2023.1301809

**Published:** 2023-10-09

**Authors:** Igor Petrušić, Peter J. Goadsby, Cristina Tassorelli, Gianluca Coppola

**Affiliations:** ^1^Laboratory for Advanced Analysis of Neuroimages, Faculty of Physical Chemistry, University of Belgrade, Belgrade, Serbia; ^2^NIHR King's Clinical Research Facility, King's College London, London, United Kingdom; ^3^Department of Neurology, University of California, Los Angeles, Los Angeles, CA, United States; ^4^Headache Science and Neurorehabilitation Centre, IRCCS C. Mondino Foundation, Pavia, Italy; ^5^Department of Brain and Behavioral Sciences, University of Pavia, Pavia, Italy; ^6^Department of Medical and Surgical Sciences and Biotechnology, Faculty of Pharmacy and Medicine, Sapienza University of Rome Polo Pontino ICOT, Latina, Italy

**Keywords:** migraine with aura, visual aura, speech symptoms, neuroimaging (anatomic and functional), headache classification, diagnosis

Typical migraine with aura (MwA) is a multifaceted primary headache disorder characterized by diverse clinical manifestations [Headache Classification Committee of the International Headache Society (IHS), 2018]. The majority of aura symptoms (roughly 90%) are visual, consisting of scintillating scotomas or fortification spectra (pure visual aura—MA) (Rasmussen and Olesen, 1992). In a subset of patients, the visual aura is accompanied by sensory or speech symptoms (visual aura plus—MA+) (Headache Classification Committee of the International Headache Society (IHS), 2018). Given that ~4.5% of the global population has experienced MwA attacks during their lifetime (Rasmussen and Olesen, 1992), it is of great importance to focus on finding new and advanced techniques for the identification and differentiation of various MwA phenotypes. This endeavor holds significant potential for enhancing the accuracy of diagnosis, classification, and identification of biomarkers specific to distinct MwA subtypes, ultimately paving the way for tailored and personalized treatment approaches for individuals suffering from MwA. Presently, such tailored treatments remain elusive due to an incomplete understanding of the complex pathophysiology underlying MwA.

It is noteworthy that throughout the course of migraine research spanning from the 20th century to the present day, the existence of MwA subtypes has not received significant attention when researchers compose comparison groups. Often, individuals with migraine, both with and without aura, or those with MA and MA+, are grouped together for research purposes. Consequently, this practice might hinder the discovery of novel biomarkers capable of unlocking new therapeutic avenues. Nevertheless, recent advances in neuroimaging and electrophysiological investigations have underscored the notable distinctions between individuals experiencing MA+ and those reporting solely visual aura preceding headache episodes (Rasmussen and Olesen, 1992; Coppola et al., 2015, 2021; Petrusic et al., 2018, 2019, 2022; Silvestro et al., 2022).

To enhance comprehension of the distinct subcategories of typical MwA within the migraine research community and expand clinical decision-making capabilities, this Research Topic aimed to uncover potential biomarkers specific to MwA subtypes and further enhance the diagnosis and stratification of distinct MwA subtypes.

Six articles have now been included in this Research Topic, containing four original research papers, one comprehensive literature review, and one meta-analysis with a systematic review.

Karsan et al. conducted an extensive literature review of neuroimaging studies in patients with typical MwA, contributing to a deeper understanding of MwA subtypes and aura pathophysiology. This topic is particularly interesting for the migraine scientific community as the migraine aura still remains an elusive phenomenon. The authors of this study suggested that migraine aura likely results from widespread brain dysfunction, affecting regions including, but not limited to, the visual cortex, somatosensory cortex, insular cortex, and thalamus. Furthermore, they highlighted that the presence of pure visual aura, as opposed to visual aura accompanied by other sensory or speech symptoms, may involve distinct functional reorganization of brain networks and additional mitochondrial dysfunction contributing to a broader spectrum of aura symptoms.

Abagnale et al. conducted significant research into structural brain changes in patients with MwA, comparing those with MA to those with MA+ presentations. The study aimed to identify microstructural abnormalities and cortical thickness variations that could differentiate between these two migraine subgroups and healthy controls. The findings revealed that MwA is associated with thinning in various cortical regions and the clinical diversity of aura symptoms is mirrored by contrasting thickness alterations in regions responsible for high-level visual processing, sensorimotor function, and language processing. While cortical thickness differences were significant, diffusivity maps did not exhibit significant variations between patients with migraine with MA or MA+ and healthy controls.

Mitrović et al. explored the morphological characteristics of the cerebral cortex in MwA patients and leveraged machine learning models for migraine detection and subtype classification using MRI data. The study employed several benchmark machine learning methods for data analysis, with linear discriminant analysis achieving a high classification accuracy of 97% for MwA patients and precise discrimination between migraine with MA and MA+, achieving an accuracy of 98%. The study identified the thickness of the pericalcarine gyrus and the left pars opercularis as the most crucial features for distinguishing between migraine with MA and MA+. This machine learning model shows significant potential in the validation of MwA diagnosis and subtype classification, which can tackle and challenge the current treatments of MwA.

Pang et al. summarized the retinal microvasculature features in patients with migraine and have provided new insights that can further our understanding of the pathological mechanisms of migraine. Their study revealed a higher prevalence of retinal and optic ischemia in migraine patients, suggesting a need for further investigations to clarify the risk of retinal and optic ischemia in individuals with MwA.

Scutelnic et al. aimed to describe the symptoms characterizing ischemic stroke and migraine aura, with a focus on their temporal evolution. The study demonstrated significant symptom overlap between ischemic stroke and migraine aura. Given the increased cerebrovascular risk in individuals with MwA, these findings hold clinical relevance and should be considered in routine medical practice, particularly when managing patients experiencing MA+ during MwA attacks.

Asawavichienjinda and Storer focused on investigating differences in treatment response between migraine patients with and without aura. Their results indicated a tendency for preventive treatment responses to be associated with specific migraine aura subtypes. Patients with complex aura presentations exhibited more favorable treatment outcomes and preventive treatment responses compared to those with only visual auras. This discovery carries significant implications; as current clinical practice typically does not differentiate treatment approaches based on the presence or absence of additional aura symptoms beyond visual disturbances.

In summary, the articles featured in this Research Topic offer a comprehensive overview of the main clinical and neuroimaging findings related to MwA and its various subtypes. Additionally, they present novel insights into this subject matter, indicating that MwA patients exhibit widespread structural cortical alterations, some of which may predispose individuals to specific MwA phenotypes. Future investigations in this field hold the potential to enhance our understanding of diverse MwA subtypes and their associated pathophysiological mechanisms, potentially culminating in the identification of innovative therapeutic targets. Furthermore, in forthcoming studies, the clinical evaluation of MwA patients should adopt a comprehensive approach in more detailed aura phenotyping, enabling the stratification of these individuals into homogeneous groups, and thus facilitating evidence-based conclusions.

## Author contributions

IP: Conceptualization, Writing—original draft, Writing—review and editing. PG: Writing—review and editing. CT: Writing—review and editing. GC: Writing—review and editing.
